# Design and Implementation of an Opioid Scorecard for Hospital System–Wide Peer Comparison of Opioid Prescribing Habits: Observational Study

**DOI:** 10.2196/44662

**Published:** 2024-09-09

**Authors:** Benjamin Heritier Slovis, Soonyip Huang, Melanie McArthur, Cara Martino, Tasia Beers, Meghan Labella, Jeffrey M Riggio, Edmund deAzevedo Pribitkin

**Affiliations:** 1 Office of Clinical Informatics, Thomas Jefferson University, 111 S 11th St, Philadelphia, PA, 19107, United States, 1 (215) 955-6000; 2 Department of Emergency Medicine, Jefferson Health Northeast, Thomas Jefferson University, Philadelphia, PA, United States; 3 Enterprise Analytics, Thomas Jefferson University, Philadelphia, PA, United States; 4 Office of Quality and Patient Safety, Thomas Jefferson University, Philadelphia, PA, United States; 5 Department of Medicine, Thomas Jefferson University, Philadelphia, PA, United States; 6 Office of the Chief Physician Executive, Thomas Jefferson University, Philadelphia, PA, United States; 7 Department of Otolaryngology, Thomas Jefferson University, Philadelphia, PA, United States

**Keywords:** opioids, peer comparison, quality, scorecard, prescribing, design, implementation, opioid, morbidity, mortality, opioid usage, opioid dependence, drug habits

## Abstract

**Background:**

Reductions in opioid prescribing by health care providers can lead to a decreased risk of opioid dependence in patients. Peer comparison has been demonstrated to impact providers’ prescribing habits, though its effect on opioid prescribing has predominantly been studied in the emergency department setting.

**Objective:**

The purpose of this study is to describe the development of an enterprise-wide opioid scorecard, the architecture of its implementation, and plans for future research on its effects.

**Methods:**

Using data generated by the author’s enterprise vendor–based electronic health record, the enterprise analytics software, and expertise from a dedicated group of informaticists, physicians, and analysts, the authors developed an opioid scorecard that was released on a quarterly basis via email to all opioid prescribers at our institution. These scorecards compare providers’ opioid prescribing habits on the basis of established metrics to those of their peers within their specialty throughout the enterprise.

**Results:**

At the time of this study’s completion, 2034 providers have received at least 1 scorecard over a 5-quarter period ending in September 2021. Poisson regression demonstrated a 1.6% quarterly reduction in opioid prescribing, and chi-square analysis demonstrated pre-post reductions in the proportion of prescriptions longer than 5 days’ duration and a morphine equivalent daily dose of >50.

**Conclusions:**

To our knowledge, this is the first peer comparison effort with high-quality evidence-based metrics of this scale published in the literature. By sharing this process for designing the metrics and the process of distribution, the authors hope to influence other health systems to attempt to curb the opioid pandemic through peer comparison. Future research examining the effects of this intervention could demonstrate significant reductions in opioid prescribing, thus potentially reducing the progression of individual patients to opioid use disorder and the associated increased risk of morbidity and mortality.

## Introduction

The United States is in an opioid epidemic originally partially fueled by legitimate but inappropriate rates of opioid prescriptions [1-4], with additional waves associated with nonprescription opioids [5]. Reducing inappropriate opioid prescribing by health care providers is a key element of mitigating the risk of long-term opioid use and potential dependence for patients [6-10]. A well-established way to combat the opioid epidemic is to reduce the number of tablets and duration of opioid therapy prescribed by health care providers [9,10]. Many guidelines have been created for this purpose; yet, prescribers routinely do not adhere to them [11,12]. Peer comparison holds the potential to improve care [13]. Research on other prescribing habits has demonstrated that when individuals identified as outliers are presented with information about how they defer from their peers, they tend to appropriately alter their prescribing habits [14,15].

The Health Information Technology for Economic and Clinical Health (HITECH) act of 2009 has made electronic health records (EHRs) ubiquitous. By 2017, 95% of US hospitals have been using EHRs [16]. EHRs and the technologies associated with them have been successfully used to combat the opioid epidemic. Electronic prescribing of controlled substances (EPCS) can improve medication safety, and a 2017 study reported that an increasing number of prescribers are prescribing electronically [17]. Prescription drug monitoring programs, which require prescribers to review prior controlled substance prescriptions prior to prescribing, have shown reductions in opioid prescribing rates [18]. Passive clinical decision support at the time of order entry has also demonstrated alterations in opioid prescribing habits [19-24], including that at Thomas Jefferson University [23,25,26].

One advantage of EHR technology is that reliable and accessible data can be used for the generation of informatics-based reports and analytics. Despite the aforementioned efforts to curb the epidemic, there remains a need for health care providers to review prescribing habits and take the initiative to improve their practice [27]. Hayes and Mycnk [27] state the following: “Better understanding of our own behavior will impact those same behaviors. Mindful practice will lead to more deliberate practice and, hopefully, improved patient care.” Though peer comparison has demonstrated positive effects with prescribing of antibiotics [14,15], research on peer comparison of opioid prescribing has been limited to the emergency department (ED) setting [28-30] and urologists [31] and has been proposed for oral maxillofacial surgeons [32]. These interventions predominantly focused on the number of prescriptions ordered and do not include other metrics such as morphine equivalent daily dose (MEDD) and how these compare to established prescribing guidelines.

In this paper, we describe the process of designing and implementing an “opioid scorecard” that demonstrates key metrics in opioid prescribing (ie, number of tablets, number of prescriptions, MEDD, and calculated day supply). These metrics include a focus on established guidelines for MEDD thresholds, previously not described in other peer comparison interventions. The thresholds for these metrics are determined at the department level, thus comparing prescribers of similar clinical backgrounds. These scorecards are released electronically on a quarterly basis and present prescribing information at the provider level, and they are distributed to individual prescribers, their department chairs, service-line leaders, and chief medical officers. Here we describe the development of the scorecard, the architecture of its implementation, and plans for future research on its effects.

## Methods

### Setting

This implementation occurred at a single hospital system: Jefferson Health (an operating division of Thomas Jefferson University). Jefferson Health includes 18 hospitals and over 50 outpatient locations across the greater Philadelphia metropolitan area. Philadelphia is a multicultural and diverse city with large Black and Hispanic populations [33]. Unfortunately, our system is partially located in the county that includes the highest estimated frequency of overdose deaths in the state [34] and contains the neighborhood cited as the source of much of the illicit trade on the East coast [35].

Jefferson Health services over 170,000 inpatient and 6.2 million outpatient visits. The system encompasses 3 divisions: the Center City Division (Thomas Jefferson University Hospital, Jefferson Hospital for Neuroscience and Methodist Hospital), Jefferson New Jersey (Washington Township, Cherry Hill and Stratford Hospitals), and the Northern Division (Abington, Lansdale, Torresdale, Bucks, and Frankford Hospitals). Within the enterprise hospital system, there are 5231 licensed prescribers, ordering over 130,000 opioid prescriptions per year. Our medical leadership noticed variability in opioid prescribing practices across the organization, prompting a need for a novel way to provide asynchronous feedback to reduce practice variation and the impetus for development of the scorecard.

### Ethical Considerations

This research was approved by the institutional review board of Thomas Jefferson University (IRB# 21E.083).

### Participants

Participants included any individual health care provider who had ordered or authorized an ambulatory prescription for an opioid medication at Jefferson Health in the last 12 months prior to the study period, regardless of division or provider specialty. Health care providers who did not order an opioid prescription in the prior 12 months were excluded.

### Defining Metrics

The author’s development group consisted of physicians, clinical informaticists, nurses, and analytics experts who are passionate about combating the opioid epidemic. Metrics were first established on the basis of prescribing guidelines developed by the Opioid Task Force (hereinafter referred to as “the task force”) for the institution, a governing body that is responsible for vetting policies and procedures for all health care providers within the health care system [36]. The task force, established in the fall of 2017, is a multidisciplinary group composed of over 40 physicians, pharmacists, nursing, and trainees who volunteer their time monthly to promote safe prescribing of opioids and promote the evidence-based management of opioid use disorder (OUD). The task force’s thresholds were influenced by recommendations from the Centers for Disease Control and Prevention (CDC) [9,10]. With this insight and guidance, the following metrics were established: (1) total number of opioid prescriptions prescribed, (2) total number of patients to whom opioids were prescribed, (3) total number of prescriptions ordered for nonchronic patients with OUD greater than a 5-day supply, (4) total number of prescriptions ordered for nonchronic patients with OUD with an MEDD greater than 50, and (5) total number of prescriptions ordered for chronic patients with OUD with an MEDD greater than 90.

### Defining the Cohort

To generate the cohort of prescribers and prescriptions, the authors first analyzed EHR data stored in the enterprise clinical data warehouse. The analysis was managed through third-party analytics software (Qlik). The authors generated a data set at the granularity of the prescription level based on various pharmaceutical classes of opioid analgesics, opioid antitussives, and their combinations for all prescriptions ordered or filled (or both) at the institution. Variables included prescriber name and unique identifier codes, the authorizing provider (if one existed for prescriptions ordered by advanced practice providers or house staff) and EHR security identifiers, patient name and identifier codes, and characteristics of the prescriptions including medication name and formulation, dosage, quantity dispensed, duration of treatment, and number of refills. The data set was stored server-side for further analysis. Through previously defined methods, the authors also generated the calculated duration of based on the number of tablets and dosage instructions for each prescription [25]. Our organization is divided into 3 divisions. Prescribers were associated with their primary division and clinical department as documented in the EHR. Providers were included in the analysis if they had ordered an opioid prescription in the last 12 months. This analysis automatically occurs quarterly on a 3-month period. The system architecture is described below.

### Calculating Thresholds

For each specialty within each division, the authors calculated the median number of opioid prescriptions ordered, the median number of patients to whom prescriptions were ordered for, and the median number of prescriptions for nonchronic patients with OUD greater than 5 days. The authors also calculated the median number of nonchronic patients with OUD per provider who had prescriptions greater than 50 MEDD and the number of chronic patients per provider for prescriptions greater than 90 MEDD. Chronic opioid use was defined as ≥3 opioid prescriptions in the last year with an active opioid prescription, which automatically includes the patient in a chronic-opioid use registry. Using the distributions of these same metrics, the authors calculated the IQRs for each specialty per division. The authors defined outlier thresholds for each specialty within the divisions as 1.5 times the IQR for the previously described metrics, and extreme outliers as 3 times the outlier threshold.

### Generating Individual Provider Reports

**Figure 1.**
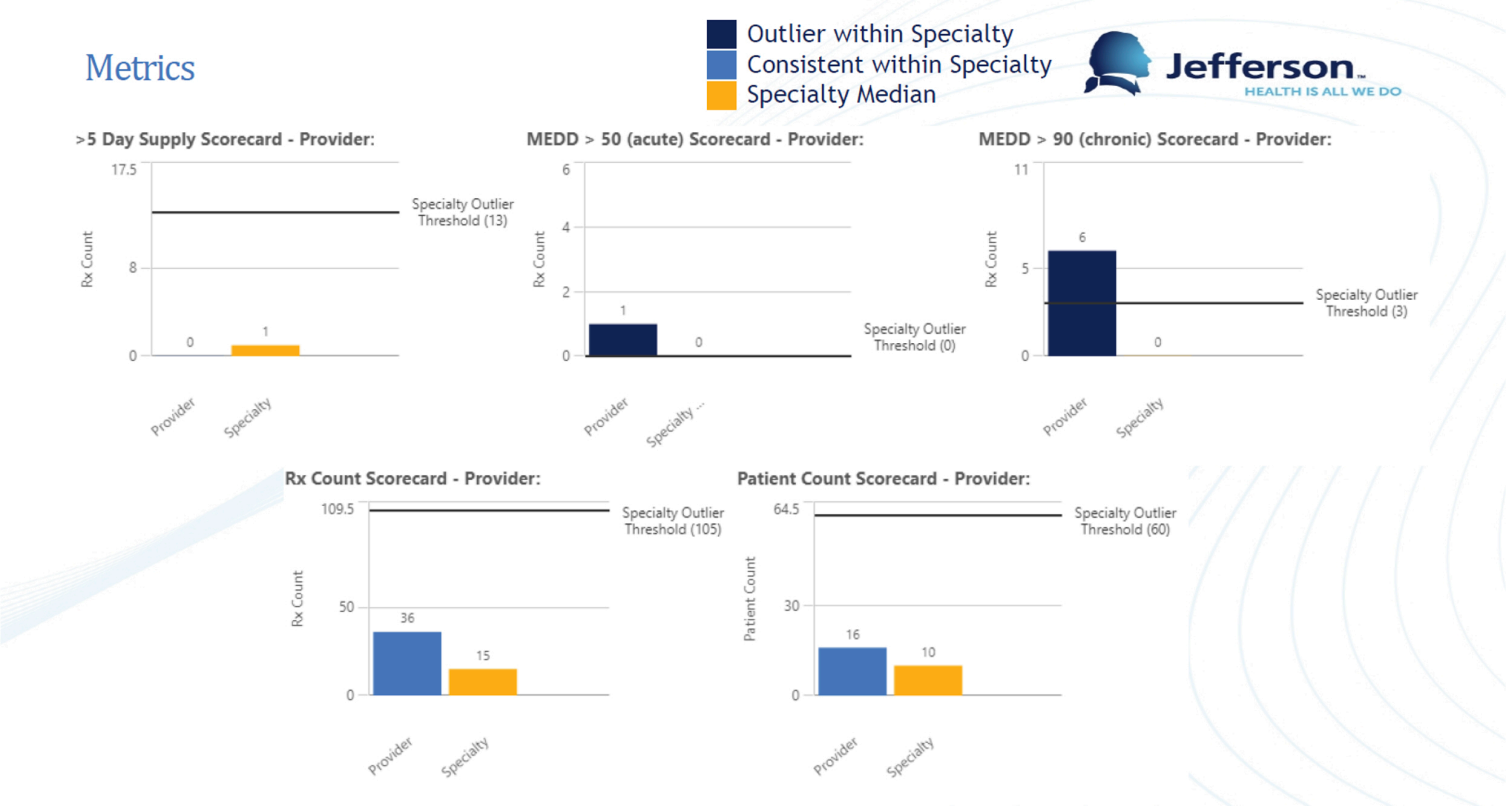
Example of a provider-level opioid scorecard representing the 5 opioid prescribing metrics, specialty metrics for comparison, and graphical representations of thresholds for that specialty. MEDD: morphine equivalent daily dose.

For individual providers, the authors calculated their values for the metrics described above. These values are compared to the calculated thresholds for outliers for their specialty within their division. If the provider is above the specialty threshold for a given metric, they are labeled an outlier. Provider-level scorecards present each of the 5 metrics as bar charts, with the value in question in the y-axis and separate bars for the provider and specialty median on the x-axis. Additionally, a line is drawn parallel to the x-axis to denote the outlier threshold. Graphical representations are color-coded: navy blue when an outlier and teal blue when consistent with their specialty for metrics with thresholds defined by the task force (greater than a 5-day supply, greater than 50 MEDD for nonchronic patients, and greater than 90 MEDD for chronic patients). The specialty medians are represented in yellow. Each scorecard contains a title page, a list of definitions, and the page containing the graphical representations of the metrics. Figure 1 demonstrates a provider score card.

### Generating Leadership Scorecards

While it is important for individual prescribers to understand their prescribing habits and perhaps reflect on and potentially modify them, it is also important for individuals in leadership positions to understand the trends in prescribing within their department. To this end, the authors created a scorecard that summarizes the prescribing habits of a department in an easy-to-interpret way with graphical representation for service-line leaders and department chairs. This allows them to discuss prescribing habits with individual prescribers and understand trends within their specialty.

A list of departmental leaders including faculty chairs and service-line leaders was compiled for the enterprise. A crosswalk was generated for each specialty to their service-line leader or chair (or both), with some leaders representing more than 1 specialty.

**Figure 2.**
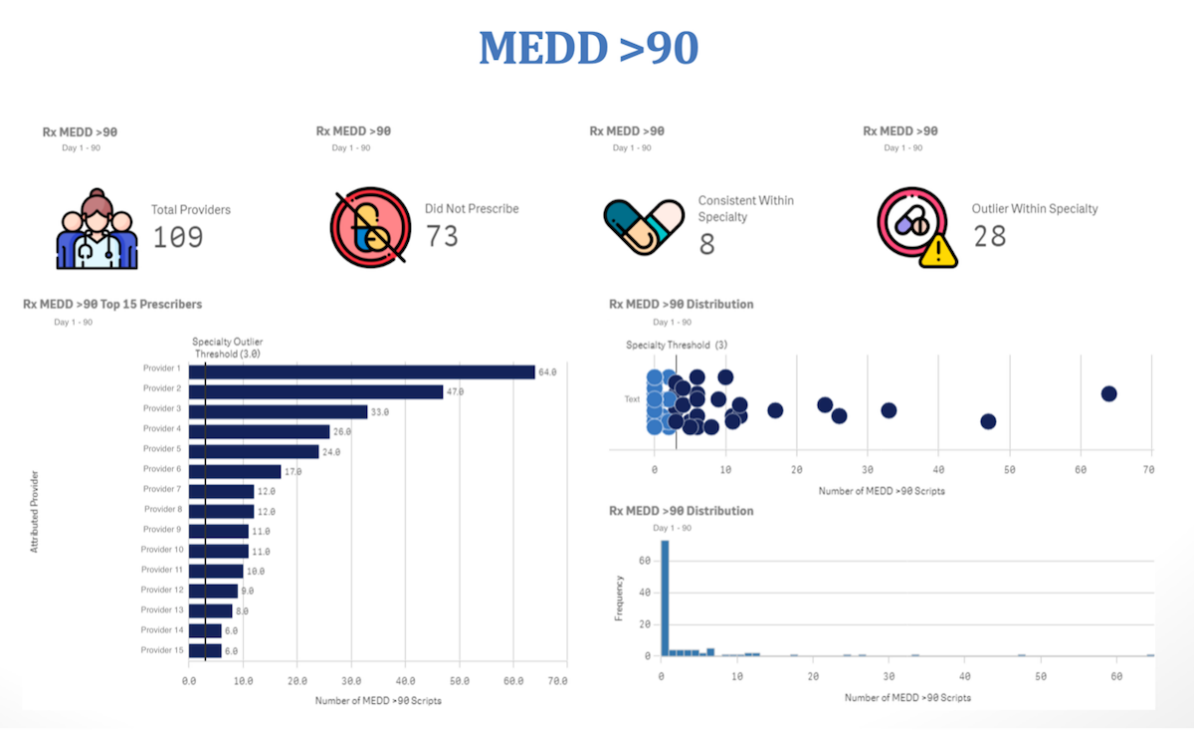
Example of a specialty leadership scorecard with described metrics and a graphical representation of top 15 prescribers by name, a color-coded scatter plot of prescribers above (dark blue) and below (light blue) specialty threshold, and a histogram of prescription frequencies.

The same metrics described in the individual provider scorecard are represented for specialty leadership with a graphical representation of the providers in their specialty. Each scorecard includes a cover letter and definitions sheet. Each metric gets a dedicated page including descriptive statistics of the number of providers included according to the criteria, the number of those who did not prescribe any opioids in that quarter, the number of those who prescribed opioids but were within the specialty threshold, and the number of individuals prescribing outside the threshold. Leaders also receive a bar chart with the name of each of the top 15 prescribers with a line on the x-axis for the specialty threshold and color-coding indicating whether each provider is within or outside the threshold. They also receive a scatter plot of all providers color-coded as under or over the threshold to better understand the distribution within their department, and a histogram of the total number of prescriptions used for the calculation of that metric. Figure 2 demonstrates a leadership scorecard example of the greater than 5-day duration metric.

### Generating Chief Medical Officer Scorecards

**Figure 3.**
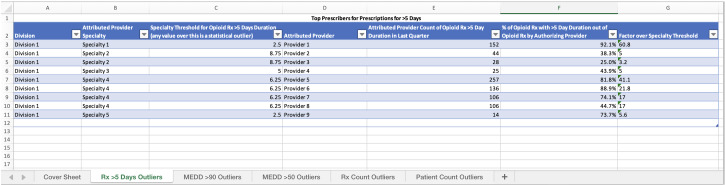
Example of the chief medical officer scorecard representing top outliers for the greater than 5 days’ supply metric. MEDD: morphine equivalent daily dose.

Given that the authors’ enterprise organizational structure has 4 separate divisions, the authors wanted to create a report that allowed division chief medical officers (CMOs) and the enterprise CMO the opportunity to comprehend and potentially interview those individuals far outside the threshold of their department. While some individuals (such as pain management or hospice care) may be appropriately prescribing outside of what a typical physician would prescribe, others may be unaware of their habits and require intervention. To this end, the authors created an automated report of individual prescribers and their metrics, which includes the previously described extreme outlier threshold set at 3 times the specialty threshold. The report is generated as an Excel (Microsoft Corp) file and distributed via email. This report is automatically shared on a quarterly basis, one for each divisional CMO representing their division and a combined summary for the enterprise CMO. An example of the CMO score card is presented in Figure 3.

### System Architecture

**Figure 4.**
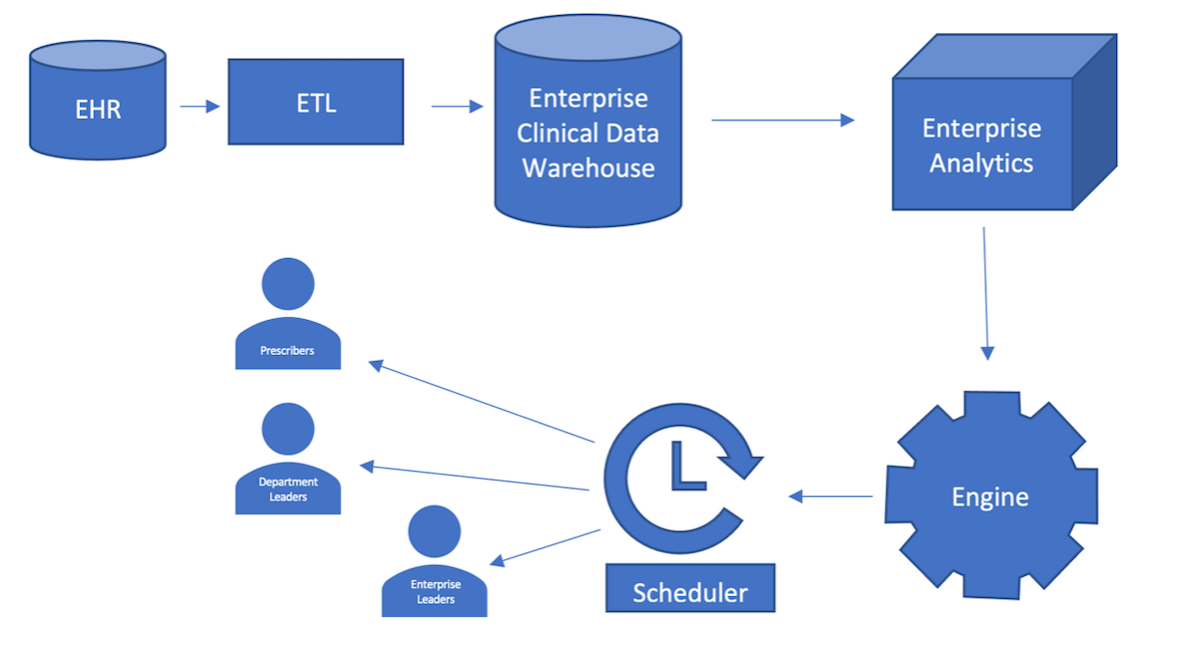
System architecture for opioid score card based on enterprise analytics workflows. EHR: electronic health record; ETL: Export, Transform, and Load.

The enterprise health system has a robust data analytics system using third-party analytics software. Data from the production EHR are extracted into a database that lives in the enterprise clinical data warehouse (CDW). We used third-party analytics software to extract and manipulate data for analysis and visualization. This third-party software uses its own proprietary language similar to SQL, which is coded by dedicated analysts from the organization’s analytics team. Through an iterative process, metrics were generated, and values were validated via the CDW query and review by clinical informatics experts. Scorecard metrics were also compared to other enterprise reports on opioids to confirm validity. Tabular and graphical representations of the data are refreshed daily and are accessible to end users via web browser. Quarterly, a report is created by the Enterprise Analytics team, through the third-party analytics software, generated automatically using the established visualizations, and is disseminated as PDF or XLS files to the appropriate recipients via secure enterprise email. Individual prescriber and leadership reports are generated as PDF files attached to individual emails with hyperlinks to the source analytics software to allow easy access by the individual recipient. There is a web-browser version of the reports that is accessible to those who have been granted access, given the sensitive nature of the reports in question. The CMO scorecards are generated as XLS files per the request of the medical leadership who wanted a quick reference report without graphical representation. Figure 4 demonstrates the system architecture.

### Implementation

The authors used a staged approach to the implementation based on access to clinical data in the CDW. Through the development of these scorecards multiple sites came upon the enterprise EHR providing a natural iterative implementation schedule but also a chance to confirm the enterprise systems could handle increasingly larger calculations and distributions of scorecards. We first piloted in the Center City division, then advancing to the New Jersey and finally the Northern division as each site generated a preimplementation 3-month period of prescribing data in the EHR, to allow for the quarterly nature of the calculations. Phased go-lives also allowed for ongoing learning and iterative improvements in these processes. Providers received an email from their divisions CMO notifying them of pending receive of the first scorecard, and each quarterly scorecard contains a cover letter from the respective CMO, but no additional education was provided outside of referring providers to their clinical leaders with additional questions. CMOs also provided information to clinical leaders about the scorecard via email prior to initial release.

### Statistical Analysis

Statistical analysis was performed using R statistical software (The R Foundation, 2021) to calculate proportions and confidence intervals. We also calculated reduction in prescription frequencies via Poisson regression analysis. To compare proportions of prescriptions outside CDC and Hospital guidelines, we performed a chi-square analysis of the first scorecard data (based on the quarter prior when no individual had ever received a scorecard) to the subsequent 4 quarters.

## Results

### Overview

A pilot version of the first scorecard was distributed in October 2019. The authors performed a staged approach to distribution, starting with the Center City Division, then expanding to New Jersey, then finally the Northern Division. Completed distribution was successful in September 2021.

To date, the authors have distributed 5 iterations of the quarterly score card over 15 months.

A total of 170 scorecards have been released to 81 service line leaders and departmental chairs over 68 departments within the 3 divisions.

Five scorecards have been released to the 4 CMOs across 3 divisions and 1 enterprise CMO.

### Prescriber Demographics

Upon the most recent distribution, which was the first to include all divisions, 2034 prescribers (38.9% of the eligible 5231 prescribers) received opioid scorecards from 68 specialties. As described above, this indicates that they have prescribed an opioid in the last 12 months, regardless of outlier status. Of these individuals, 1416 (69.7%) were attending physicians, 331 (16.3%) were nurse practitioners, 168 (8.3%) were physician assistants, and 111 (5.5%) were residents with independent Drug Enforcement Administration licenses.

### Outlier Demographics

There were 386 (19.0% of 2034 opioid prescribers, 7.31% of the 5231 licensed prescribers) individuals who were considered outliers in their specialty for at least 1 metric. Of them, 188 (48.7%) were outliers for total number of prescriptions, 162 (42.0%) were outliers for the number of patients prescribed opioids, 125 (32.4%) were outliers for prescriptions ordered for nonchronic patients with prescriptions greater than 5 days, 118 (30.6%) were outliers for prescriptions greater than 50 MEDD for unique nonchronic patients and 113 (29.3%) were outliers for prescriptions greater than 90 MEDD for chronic patients.

Table 1 demonstrates cumulative counts and percentages of outliers from the first 5 scorecards released for each of the 5 metrics from the 5 largest clinical specialties in the organization, deidentified to protect participating physicians. Notably, at least 10% of providers were considered outliers in at least 1 metric during the initial 5 scorecard releases.

**Table 1.** Cumulative counts and percentages of outliers from the first 5 scorecards released for each of the 5 metrics from the 5 largest clinical specialties in the organization.

Specialties	Providers, n	Rx count outliers, n (%)	Patient count outliers, n (%)	>5 day supply outliers, n (%)	>50 MEDD^a^ outliers, n (%)	>90 MEDD outliers, n (%)
1	212	33 (15.57)	35 (16.51)	42 (19.81)	60 (28.30)	6 (2.83)
2	195	26 (12.26)	28 (13.21)	63 (29.72)	41 (19.34)	39 (18.40)
3	176	19 (8.96)	11 (5.19)	26 (12.26)	51 (24.06)	50 (23.58)
4	101	8 (3.77)	10 (4.72)	42 (19.81)	33 (15.57)	11 (5.19)
5	97	9 (4.25)	7 (3.3)	10 (4.72)	18 (8.49)	15 (7.08)

a MEDD: morphine equivalent daily dose.

### Prescribing Impact

We examined the iterative scorecard releases to determine if there was an effect on opioid prescribing. Given the phased implementation process, the Center City Division was the only division of the organization with a full 5 quarters of scorecard releases, so this division was isolated for analysis. There were 2,147,710 opioid prescriptions ordered by this division during the study period. From the date of initial release, there was a significant reduction in the frequency of opioid prescriptions at the division, with an associated 1.60% reduction per quarter (*P*<.001). There was a significant reduction in prescriptions for nonchronic patients longer than 5 days’ duration before versus after intervention. There was also a significant reduction in the proportion of prescriptions >50 MEDD for nonchronic patients and an increase in that of prescriptions >90 MEDD for chronic patients (Table 2).

**Table 2.** Pre- and postintervention proportions, 95% CIs, and *P* values for >5-day durations, >50 MEDD^a^ for nonchronic patients with opioid use, and >90 MEDD for chronic patients with opioid use^b^.

Study period	>5-days duration		>50 MEDD for nonchronic patients		>90 MEDD for chronic patients	
	Proportion, %	95% CI	Proportion, %	95% CI	Proportion, %	95% CI
Pre	27.31	27.18‐27.43	13.06	12.96‐13.16	6.64	6.56‐6.71
Post	26.76	26.69‐26.82	12.14	12.04‐12.24	6.80	6.76‐6.84

a MEDD: morphine equivalent daily dose.

b *P*<.001 for all pre- vs postintervention comparisons.

## Discussion

### Principal Findings

The objective of this study is to describe an evidence-based peer comparison opioid scorecard. By sharing this process of design and distribution, we hope to inspire similar processes at other organizations. Here we demonstrate the successful distribution of scorecards to 19% (386/2034) of eligible opioid prescribers over a 5-quarter time frame. Initial analysis demonstrates that in all 5 of the largest specialties, at least 10% of providers were outliers in at least 1 metric (Table 1). This is most notable in specialties 1 and 3 where over 20% of providers had prescriptions greater than 50 MEDD for nonchronic patients, and over 19% of providers in specialties 1, 2, and 4 had prescriptions over 5 days’ supply for nonchronic patients. These results demonstrate the need for intervention and providing information to individual providers on their prescribing practices at the organization.

Over the study period, the authors demonstrated a quarterly reduction in overall opioid prescriptions of 1.6% per quarter. When comparing the data that were used to generate the initial scorecard (the quarter prior to scorecard release) versus the subsequent 4 quarters, there was a small but significant reduction in the proportion of prescriptions longer than 5 days’ duration and greater than 50 MEDD for nonchronic patients (0.6% and 0.3%, respectively). While the proportions were small overall, the median quarterly prescribing rate at this division was over 420,000 prescriptions, yielding a quarterly reduction of over 2500 prescriptions over 5 days and 1200 prescriptions over 50 MEDD. There was also a very small but significant increase in chronic patient prescriptions greater than 90 MEDD (0.16%), resulting in approximate 672 additional patients with this level of MEDD per quarter. Given the nature of this study, it is unclear if these are patients who transitioned from nonchronic to chronic dosage changes, or if this change was due to other external factors that influenced prescribing habits.

Since the early 1990s, opioid prescribing has consistently increased [37] to the point that in 2016, there were 66.5 opioid prescriptions for every 100 persons in the United States [38]. There is a link between long-term use of prescription opioids and a transition to illicit heroin [39] with increasing mortality [40]. Higher doses and longer treatment time of prescription opioids have been linked to an increased risk of chronic opioid use [6-8]. Therefore, a principal method of combating the opioid epidemic is to reduce prescribing by health care providers. To this end, the CDC’s 2016 guidelines recommend that caution be exercised when increasing doses to greater than 50 MEDD, and doses greater than 90 MEDD should be avoided. The CDC also states that 3 days or less is often sufficient for acute pain, and more than 7 days is “rarely needed” [9,10]. Our organization has accordingly recommended maintaining prescriptions for acute pain at 5 days and less than 50 MEDD, based on these guidelines. The inclusion of these evidence-based metrics in the scorecard justifies its evidence-based nature.

Peer comparison has been demonstrated to be an effective means of modifying unwanted or unintended behavior among health care providers [14,15,28-31]. Andereck et al [28] and Boyle et al [29] both demonstrated reductions in prescribing rates after peer comparison reports were initiated in their EDs. Michael et al [30] performed a randomized controlled trial of ED physicians and demonstrated a relationship between the perceived rate of prescribing and a reduction when presented with peer comparison data. Suffoletto and Landaou [41] demonstrated that peer comparison was an important contributor to improved prescribing habits compared to audit and feedback alone. While limited to the ED, these research studies justify our approach to peer comparison and confirm that prescriber’s own perceptions of their prescribing habits are critical to successful intervention.

Research beyond the ED is limited regarding peer comparison of opioid prescribing. Jacobs et al [31] demonstrated that peer comparison, as well as education and audit feedback, contributed to significant reductions in prescribing by urologists. Weiner et al [42] describe a system-wide initiative that successfully reduced opioid-related morbidity and mortality. They include a mention of “benchmarking reports” with peer comparison elements but do not further detail what was included in these reports or how they were delivered.

Despite evidence of its positive impact on prescribing, a report such as the one presented in this paper has never been produced outside of isolated EDs nor has it been implemented as broadly. The intervention described here is sent to all prescribers, regardless of level of training or clinical specialty, providing them with detailed metrics on their opioid prescribing habits and peer comparison based on clinical specialty. Additionally, most of the previously described department-level interventions focus on opioid prescribing frequency without consideration toward prescribing guidelines such as those recommended by the CDC. The only other system-wide effort described in the literature contains limited information on what was included in their reports, how information was portrayed, and how they were delivered, but rather focused more on other elements of their substantial and impressive system-wide intervention.

The methods described in this paper have the potential to change clinical practice paradigms by using a novel intervention implemented at a larger scale than previously described in the literature. While peer comparison has been used previously in this manner (as described above), little is known about how such a tool could be used on an enterprise hospital level, with multiple divisions encompassing multiple practice specialties across 2 states. As longer and higher doses of opioids are associated with long-term use and potential for OUD, educating prescribers on their opioid prescribing habits can potentially reduce the prescription quantity and duration at the prescriber level, thus potentially mitigating the risk of long-term use at the patient level.

The scorecards received by prescribers contain multiple metrics including the number of prescriptions, number of patients, and MEDD thresholds. Outlier thresholds based off IQRs are calculated quarterly. We appreciate that overtime variability in prescribing patterns may reduce, resulting in lower thresholds that are incapable of driving impact. Currently, all scorecards are delivered with instructions on how the metrics are derived, and they are informational and nonpunitive in nature. Providers are instructed that concerns regarding metrics should be discussed with departmental chairs or service line leaders to determine whether intervention is required. Reinforcement of appropriate prescribing guidelines by leaders was not systematically carried out; however, such systematic enforcement holds promise for further reduction in inappropriate prescribing.

The authors intend to continue to prospectively track the metrics associated with each scorecard release. By measuring descriptive statistics for each specialty to determine whether there are changes in that specialty’s prescribing habits before and after intervention, as well as through a time series post intervention, the authors hope to demonstrate the impact that the scorecard and enterprise-level peer comparison can have on opioid prescribing beyond the initial results presented here. The authors will also evaluate frequencies with which individual prescribers are identified as outliers and track any changes in metrics of individual high-prescribing providers. The authors are also tracking quarterly thresholds as a measure of improvement in practice variation and to determine whether prolonged periods of stasis imply that no further reduction in practice pattern variability can be achieved.

Given this effective implementation, positive results could allow for broadening of the scope of the study and for massive scalability. The system described is EHR-agnostic given the use of third-party reporting software, meaning that data sources from other hospital systems could be integrated and similar reports can be generated on a regional, state, or even federal level.

Additionally, while this project is intended to address opioid prescribing for which clear practice guidelines have been established by the CDC, the application of automated peer comparison reports and assessment of their effects on practice patterns can be extended to topics including but not limited to antibiotic prescribing, imaging usage, laboratory test ordering, and specialty consultation. Automated peer comparison could have the potential to reduce cost and improve the quality of care when designed around appropriately established clinical guidelines.

### Limitations

There are several limitations that should be addressed. Our study was performed at a single hospital system, and it is unclear if our intervention would have similar effects at institutions dissimilar to ours in different geographical regions. Additionally, we cannot account for all confounders that may have been contributed to reductions in opioid prescribing outside of our intervention. Our organization has historically used multiple interventions to curb the opioid epidemic including but not limited to requiring the review of state prescription drug monitoring programs prior to outpatient prescribing and standardized dosing for opioids in our EHR. However, there were no concurrent internal interventions during the time of the implementation, though external influences such as state and regional educational efforts could have impacted prescribing. As mentioned above, we also cannot account for possible transitions of patients from nonchronic to chronic cohorts during the study period, which may have contributed to the small increase in nonchronic prescribing, though it is also unclear if external factors may have contributed to this finding. Additionally, with regard to prescriptions, we only examined trends in opioid prescribing and did not examine whether there were any contrary changes in other nonopioid medications as an unintended consequence of this intervention. Finally, in late 2022, the CDC updated their opioid prescribing guidelines, which have transitioned from the specific acute and chronic thresholds for MEDD to more general recommendations on prescribing the lowest dose of opioids possible to achieve pain control, with detailed information on the risks of doses above 50 MEDD [43]. The intervention detailed here was developed for the 2016 guidelines, and our organization still supports the prescribing thresholds developed from them. Modifications to this intervention will be developed while pending institutional transitions to the new prescribing guidelines.

### Conclusions

Modern EHR technology, advanced analytics, and a robust reporting infrastructure allowed for the generation of an enterprise-level opioid prescribing scorecard that uses peer comparison to provide individual providers with feedback on their prescribing habits. The authors also have successfully developed departmental leadership and CMO reports for oversight and advanced intervention regarding potentially problematic prescribers. Initial results over a 5-quarter period imply reductions in overall opioid prescribing rates as well as improvement in the duration and dose for nonchronic patients, though there was a small increase in prescriptions over 90 MEDD. Future research on the impact of such a scorecard could be pivotal in combating the opioid epidemic, potentially scaling such an intervention to larger geographical regions, and broadening the use of such a tool outside the opioid epidemic. Investment in informatics and analytics holds the potential to have profound impacts on the quality of care and patient safety.

## Abbreviations

CDC: Centers for Disease Control and Prevention
CDW: clinical data warehouse
CMO: chief medical officer
ED: emergency department
EHR: electronic health record
EPCS: electronic prescribing of controlled substances
HITECH: Health Information Technology for Economic and Clinical Health
MEDD: morphine equivalent daily dose
OUD: opioid use disorder
